# Impacts of skull stripping on construction of three-dimensional T1-weighted imaging-based brain structural network in full-term neonates

**DOI:** 10.1186/s12938-020-00785-0

**Published:** 2020-06-03

**Authors:** Geliang Wang, Yajie Hu, Xianjun Li, Miaomiao Wang, Congcong Liu, Jian Yang, Chao Jin

**Affiliations:** Department of Radiology, The First Affiliated Hospital of Xi’an Jiaotong University, Xi’an, 710061 People’s Republic of China

**Keywords:** Skull striping, Neonatal brain, Brain structural network, 3D T1-weighted imaging

## Abstract

**Background:**

Skull stripping remains a challenge for neonatal brain MR image analysis. However, little is known about the accuracy of how skull stripping affects the neonatal brain tissue segmentation and subsequent network construction. This paper therefore aimed to clarify this issue by comparing two automatic (FMRIB Software Library’s Brain Extraction Tool, BET; Infant Brain Extraction and Analysis Toolbox, iBEAT) and a semiautomatic (iBEAT with manual correction) processes in constructing 3D T1-weighted imaging (T1WI)-based brain structural network.

**Methods:**

Twenty-two full-term neonates (gestational age, 37–42 weeks; boys/girls, 13/9) without abnormalities on MRI who underwent brain 3D T1WI were retrospectively recruited. Two automatic (BET and iBEAT) and a semiautomatic preprocessing (iBEAT with manual correction) workflows were separately used to perform the skull stripping. Brain tissue segmentation and volume calculation were performed by a Johns Hopkins atlas-based method. Sixty-four gray matter regions were selected as nodes; volume covariance network and its properties (clustering coefficient, *C*_*p*_; characteristic path length, *L*_*p*_; local efficiency, *E*_local_; global efficiency, *E*_global_) were calculated by GRETNA. Analysis of variance (ANOVA) was used to compare the differences in the calculated volume between three workflows.

**Results:**

There were significant differences in volumes of 50 brain regions between the three workflows (*P *< 0.05). Three neonatal brain structural networks presented small-world topology. The semiautomatic workflow showed remarkably decreased C_*p*_, increased L_*p*_, decreased *E*_local_, and decreased *E*_global_, in contrast to the two automatic ones.

**Conclusions:**

Imperfect skull stripping indeed affected the accuracy of brain structural network in full-term neonates.

## Background

It is known that magnetic resonance imaging (MRI) has become a very important tool for investigating the early brain development and injury in neonates [1–3]. In particular, MRI-based brain structural network analysis provides critical ways to understand the topological structure of brain information integration and transmission during the early development [4–7]. As a basic preprocessing, skull stripping, designed to eliminate skull, scalp, dura, and other non-brain tissues and retain brain parenchyma from brain MRI, is an essential process in brain tissue segmentation and subsequent brain network construction [8]. For neonates, numerous efforts has been made to perform the automated processing [9–18], such as FMRIB Software Library’s (FSL) Brain Extraction Tool (BET) [19], infant brain extraction and analysis toolbox (iBEAT developed by the IDEA group at the University of North Carolina at Chapel Hill) [20]. In detail, BET performed the brain extraction by using a deformable surface model to detect the brain boundaries, and the results highly depend on the used parameters [19]. Differently, a learning-based method which combines a meta-algorithm and level-set fusion has been employed in iBEAT [20]. iBEAT shows good performance in infant skull stripping with extensive evaluations on more than 200 infants. Despite this, skull stripping remains a challenge for neonatal brain MRI analysis due to its low tissue contrast, large within-tissue intensity variations, and regionally heterogeneous [21]. Besides, inaccurate skull stripping, e.g., unremoved non-brain tissues would result in the overestimation of local brain volume and cortical thickness [22]. And it would further affect the construction of brain structural network. To our knowledge, little is known about the impact of preprocessing accuracy on the accuracy of brain tissue segmentation and structural network construction.

The present study aimed to investigate the effects of skull stripping on brain tissue segmentation and structural network construction based on three-dimensional (3D) T1-weighted imaging (T1WI). Firstly, we constructed the processing flow based on 3D T1WI on brain gray matter in 22 term neonates; secondly, repeatability and consistency of brain region volume’s segmentation were performed to verify the accuracy; in final part, the volumes of 64 brain region and properties of brain structural network were calculated and compared between three workflows, i.e., two automatic (BET, iBEAT) and a semiautomatic iBEAT with manual correction. Such a workflow could be applied to characterize brain structural connectivity and may provide valuable anticipatory information about the potential for encountering abnormalities at a later stage in development.

This paper is organized as follows. In “Results” section, we report the results on repeatability and reliability, and comparisons of brain structural network between automatic and semiautomatic workflows. In “Discussion” section, we provide additional discussions and followed by conclusion in “Conclusion” section. The methods for repeatability and reliability test, and construction of brain structural network were provided in “Methods” section.

## Results

### Repeatability and reliability

**Fig. 1:**
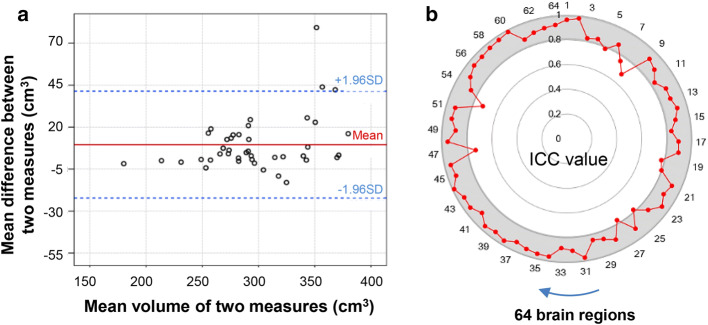
Results of repeatability and reliability of brain region volume calculated by iBEAT with correction. **a** Bland–Altman graph of whole brain volume between two measurements with 95% of the differences located in the mean ± 1.96 SD (standard deviation) range showing the good repeatability; **b** intraclass correlation coefficient (ICC) of volume of 64 brain regions between two repeated measurements

The Bland–Altman graph of two repeated measurements indicated that 95% of the differences between two measurements located in the mean ± 1.96 SD (standard deviation) range that suggested good repeatability (Fig. 1a). Meanwhile, the ICC analysis indicated the average correlation coefficient was 0.894. Most of values were greater than 0.8, while those of a few brain regions, e.g., caudate nucleus, precuneus, superior occipital gyrus, and inferior occipital gyrus were relatively lower, but were greater than 0.7 (Fig. 1b).

### Comparisons of brain structural network between three workflows

#### Brain region volume

**Fig. 2:**
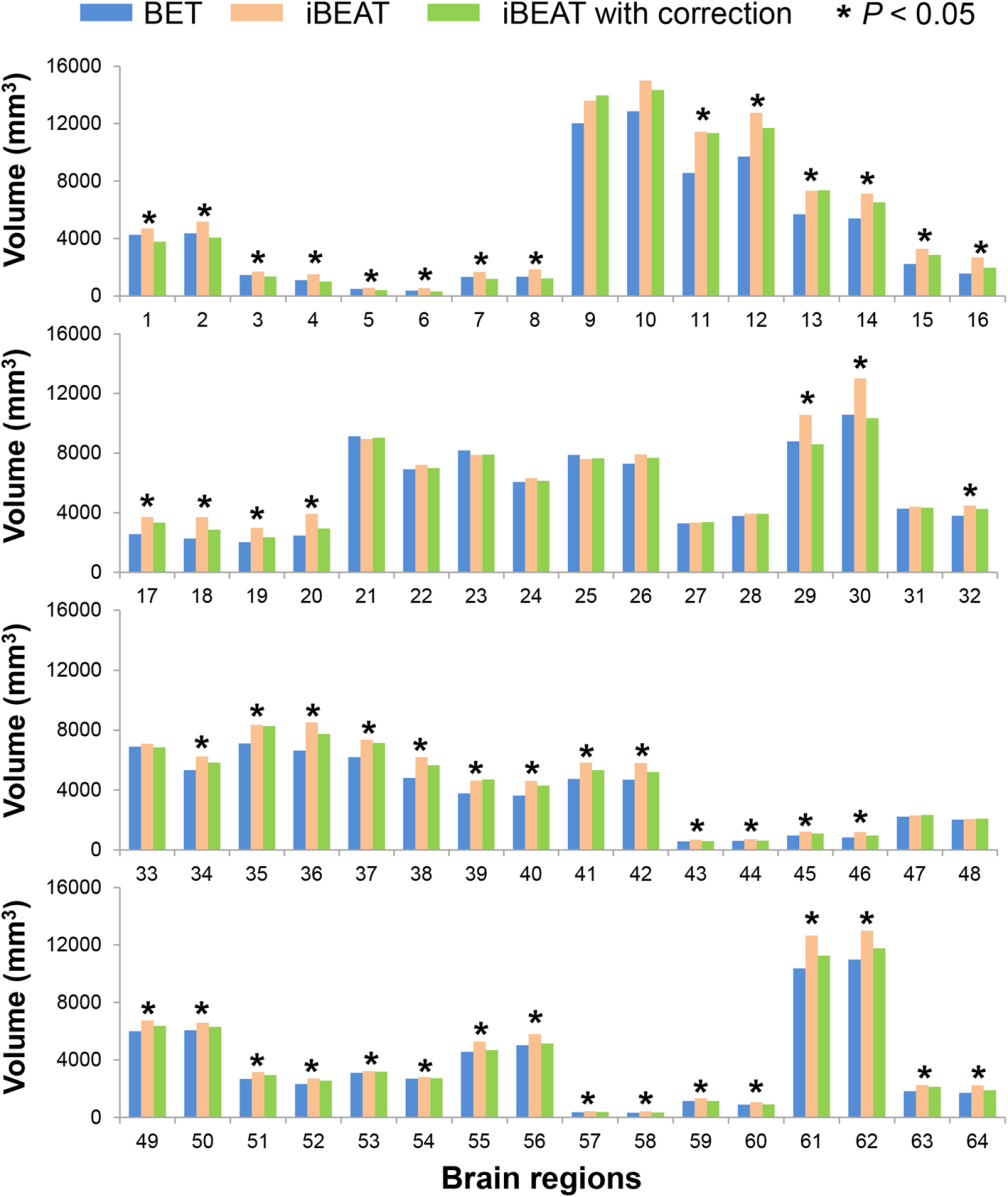
Comparisons of the calculated volume of 64 brain regions between BET, iBEAT and iBEAT with correction by analysis of variance (ANOVA)

The differences of volume of 64 brain regions performed through ANOVA analysis among the three workflows are shown below (Fig. 2). There were significant differences of volume between three workflows in 50 brain regions, while, no significant difference was found in the remaining 14 brain regions, such as bilateral superior frontal gyrus, bilateral pre- and post-central gyrus, bilateral superior parietal gyrus, bilateral precuneus, bilateral superior occipital gyrus and so on.

#### Small-world properties

**Fig. 3:**
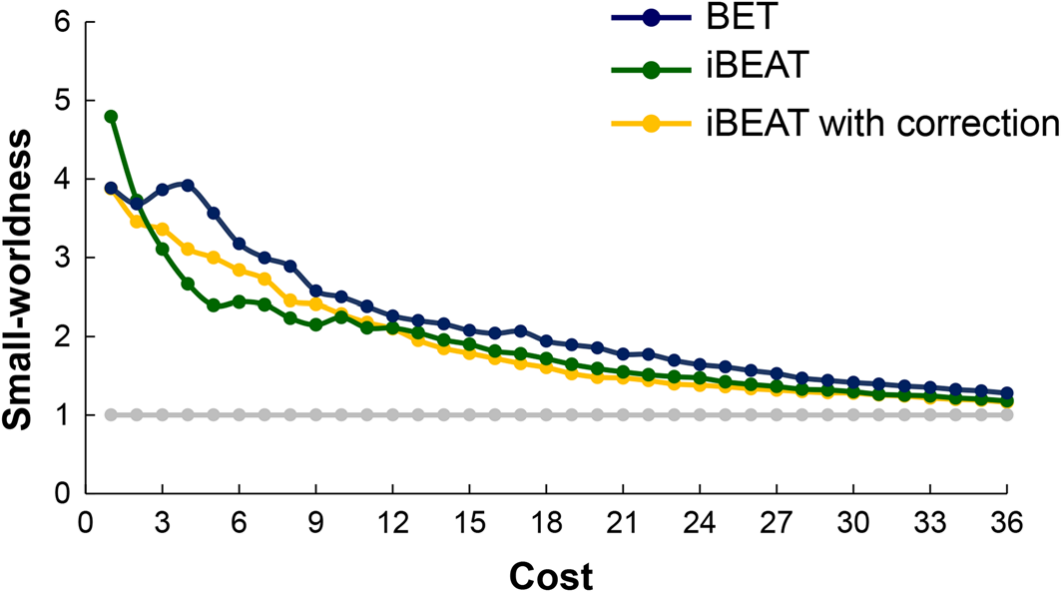
Comparisons of small-world property of brain structural network between BET, iBEAT and iBEAT with correction. A small-world network should fulfill the following conditions: *C*_*p*_/*C*_rand_ > 1 and *L*_*p*_/*L*_rand_ ≈ 1; *C*_*p*_ and *L*_*p*_ indicate the clustering coefficient and characteristic path length, respectively

In the defined threshold range, the neonatal brain network exhibited high-efficiency small-world topology (Fig. 3).

#### Properties of brain structural network

**Fig. 4:**
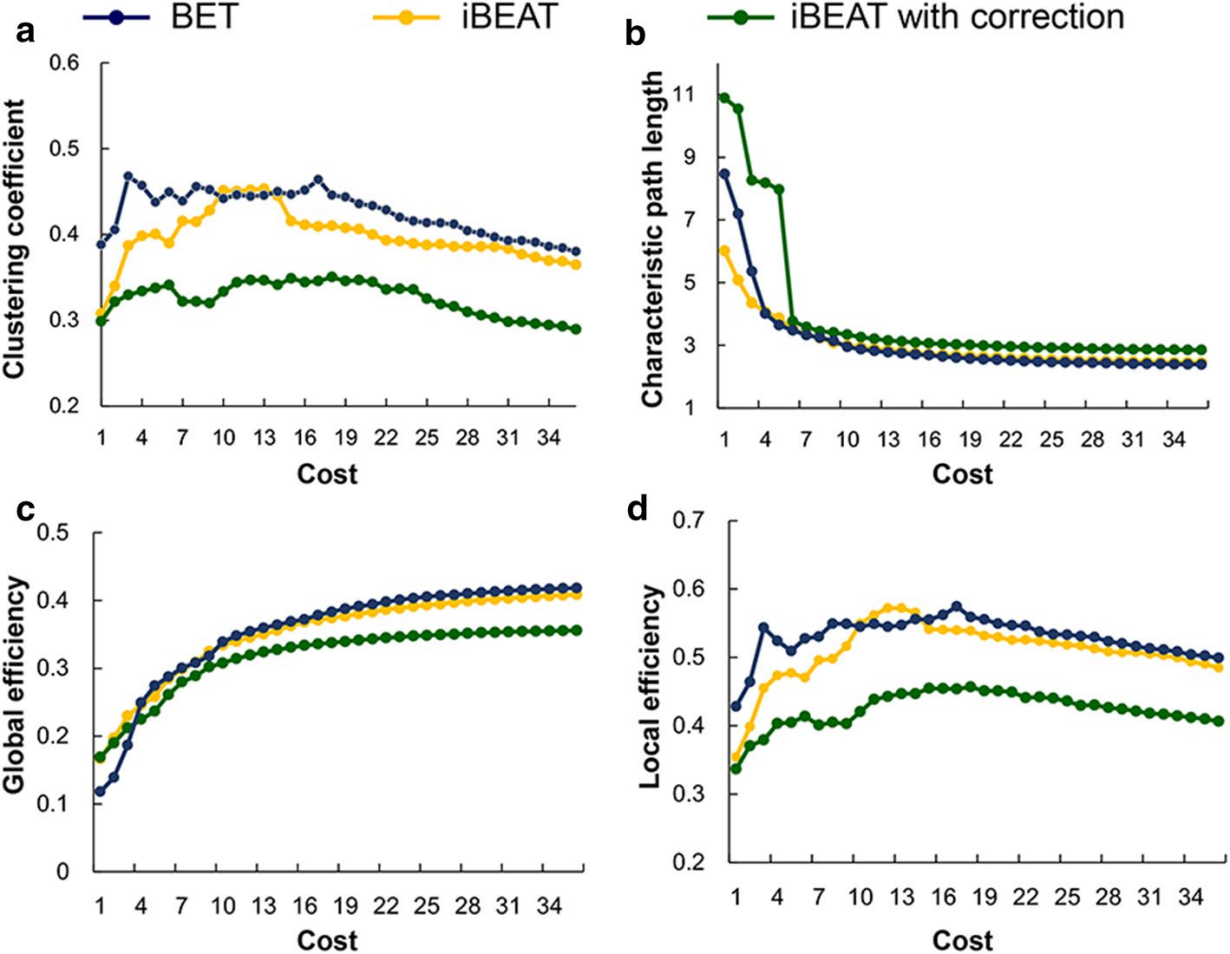
Comparisons of properties of brain structural network between BET, iBEAT and iBEAT with correction. **a** clustering coefficient, **b** characteristic path length, **c** global efficiency, **d** local efficiency

The corrected workflow, compared with the others, showed a significantly decreased *C*_*p*_ and increased *L*_*p*_. With regard to network efficiency, the corrected workflow showed a significantly decreased *E*_local_ and *E*_global_ (Fig. 4).

## Discussion

To investigate the impacts of skull stripping on the accuracy of brain tissue segmentation and structural network construction, three workflows (BET, iBEAT and iBEAT with manual correction) were compared to perform the 3D T1WI-based brain structural network. Our results indicated that the iBEAT with manual correction showed a more accurate consistency and repeatability in brain segmentation. Besides, significant differences in calculations of brain volume and structural network properties between the three workflows further implied the importance of accurate skull stripping in brain segmentation and subsequent brain network construction.

The ICC results indicated that the majority of brain regions showed good repeatability and reliability, while the ICC of some regions, such as caudate nucleus, gyrus rectus, left superior parietal gyrus, left precuneus, left superior occipital gyrus and right inferior occipital gyrus, were relatively lower. One reason may be rooted in the inherently low spatial resolution, insufficient tissue contrast, and ambiguous tissue intensity distributions in neonatal MRI [21]. On the other hand, the adult head coil used in this study may affect the MR image quality in neonates [23, 24]. In this regard, MRI acquisition settings including the specific head coil and scanning parameters should be adjusted for neonates. In detail, a neonatal head coil with appropriate size and high signal-to-noise ratio, as well as the use of appropriate scanning parameters (e.g., smaller FOV, longer repetition time and echo time than adult scanning) would facilitate the signal acquisition [23, 25].

**Fig. 5:**
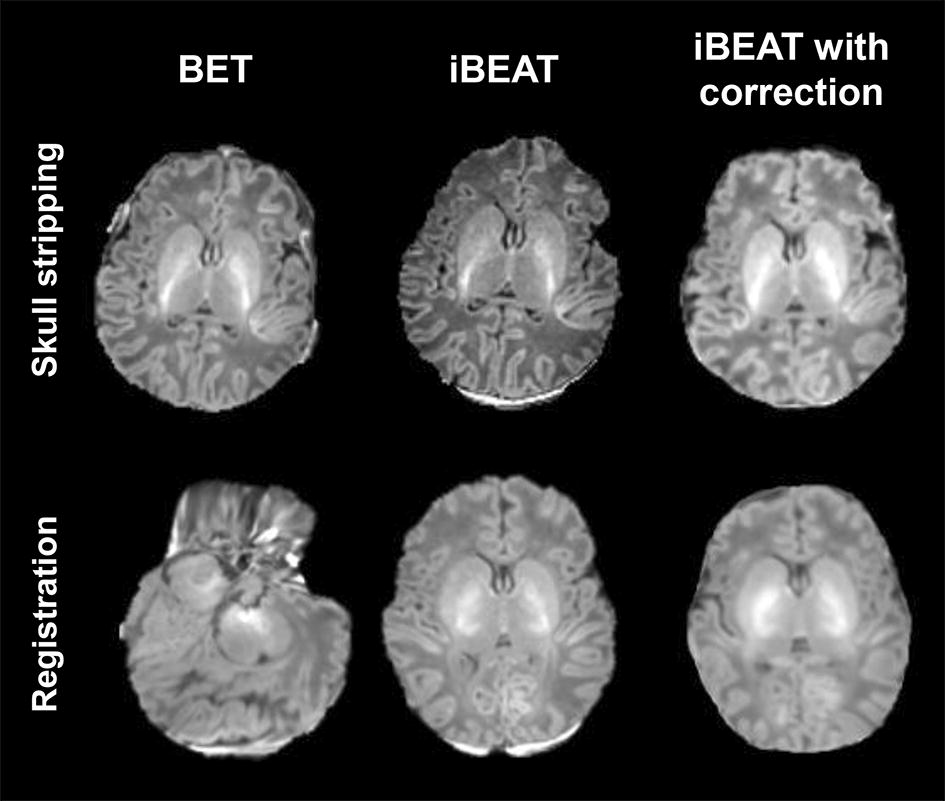
Illustrations of skull stripping and registration by BET, iBEAT and iBEAT with manual correction

By comparing the three workflows, we found that slight adjustment for skull stripping would remarkably affect the calculations of regional brain volume. Of 64 regions, significant differences existed in brain volume calculations of 50 regions between the three workflows. This further confirmed the difficulty of brain segmentation in neonatal MRI. Specifically, most brain volumes calculated by BET workflow were smaller than those by iBEAT and iBEAT with manual correction. This may be linked to the difference of skull stripping in neonatal MRI between BET and iBEAT [21]. It is worth noting that more unremoved skull components were found in BET than iBEAT; while unremoved components by iBEAT were mainly located in the base of skull (Fig. 5). It may be such facts that led to the larger brain volumes by iBEAT than iBEAT with manual correction. From the above, the accuracy of skull stripping would have considerable effects on the neonatal brain segmentation.

By studying the brain structural network, we found the highly small-world topology in full-term neonates. This may suggest that a highly efficient brain network, serving for brain information integration and transfer, has been constructed in the early development. This is also in agreement with prior findings in neonates [7]. Besides, significant differences were found in local (C_*p*_ and *E*_local_) and global (*E*_global_) topological properties of brain structural network between the three workflows. These may indicate that these parameters were sensitive to the accuracy of preprocessing (skull stripping).

The study had several limitations. First, the sample size was relatively small in this study, and more data should be acquired to further improve the accuracy. Second, the template and atlas we used are made by foreign neonates. Given the differences of brain morphology between Western and Eastern neonates [26–29], it may lead to certain errors in the initial estimation of tissue intensity distributions and thus resulted in the large deformations in the registration procedure. In this regard, a dedicated atlas and template appropriate for Chinese neonates need to be developed in further study. Besides, based on the above, further studies regarding the differences of brain structural network constructed by iBEAT and iBEAT with manual correction between Western and Eastern neonates are also required to facilitate the understandings of preprocessing’s impacts on the brain network construction. Third, skull stripping by the time-consuming semi-automated workflow, i.e., iBEAT with manual correction was implemented; an automated method is further required to perform a more accurate skull stripping.

## Conclusions

In this study, we quantitatively analyzed the influences of skull stripping on brain volume calculation and topological properties of brain structural network in neonates. Our results indicated that the brain networks had robust small-world configuration; in addition, there were significant differences in both local and global topological parameters between the three workflows. These findings enhanced the importance of accurate skull stripping in calculation of brain tissue volume and brain structural network construction.

## Methods

This study was approved by the local institutional review board of the first author’s affiliation. The parents of the neonates were informed regarding the goals and risks of the MRI scan, and requested for the written consent.

### Subjects

This study recruited 22 full-term neonates (13 males and 9 females; gestational age range: 37–42 weeks) without any MRI abnormalities or evidences of any clinical episodes that might cause cerebral damages.

### MRI data acquisition

All data were acquired on a 3.0-Tesla scanner (Signa HDxt, General Electric Medical System, Milwaukee, WI, USA) with an 8-channel phase array radio-frequency head coil. To reduce the head movement and complete the MRI procedure, the subjects were sedated with a relatively low dose of oral chloral hydrate (25–50 mg/kg). The potential risks of the chloral hydrate were fully considered. The selection, monitoring, and management of subjects were strictly performed following the guidelines [30]. Neonates were laid in a supine position and snugly swaddled in blankets. A pediatrician was present during the MRI scan. Micro-earplugs were inserted into the external auditory canal for hearing protection. Heads of the subjects were immobilized by molded foam, which was placed around the head. The temperature, heart rate, and oxygen saturation were monitored throughout the procedure.

Three-dimensional fast spoiled gradient-recalled echo (3D-FSPGR) T1-weighted magnetic resonance images were acquired with the parameters: repetition time/echo time = 10.28 ms/4.62 ms, inversion time = 400 ms, field of view = 240 mm, voxel size = 0.94 × 0.94 × 1 mm^3^.

### Construction of brain structural network

**Fig. 6:**
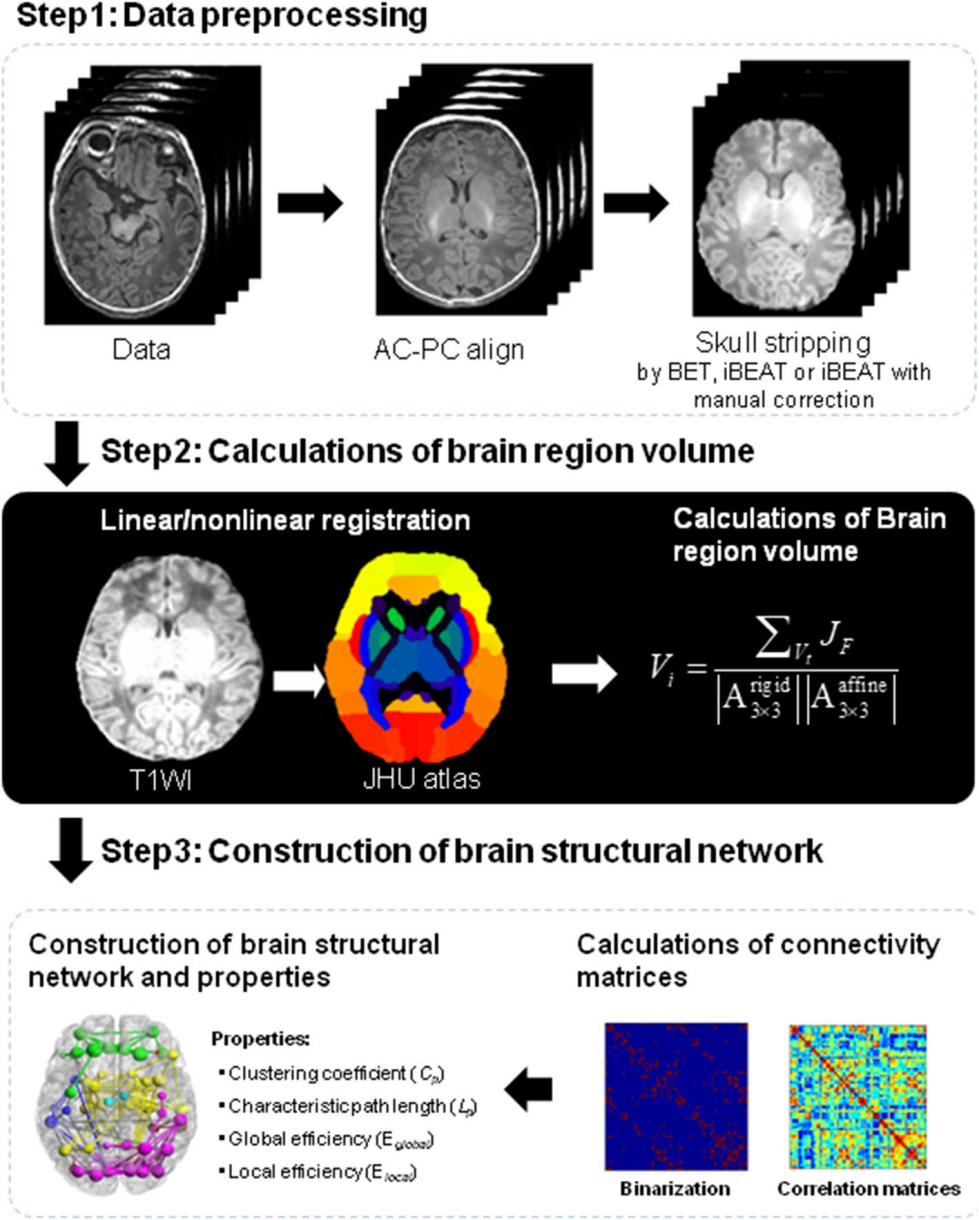
Workflow for construction of neonatal brain structural network. AC–PC align refers to the alignment of MRI data according to anterior commissure–posterior commissure line

Figure 6 provides the workflow for constructing the neonatal brain structural network. The three steps for the workflow were detailed as follows.

#### Data preprocessing

To investigate the impacts of data preprocessing on network construction, three preprocessing workflows were performed by BET, iBEAT and iBEAT with manual correction.

BET workflow: non-brain tissues in each T1WI image were automatically removed by BET. iBEAT workflow: three automatic preprocessing steps, including brain contrast enhancement and skull stripping were sequentially performed. iBEAT with manual correction: (1) manually align the AC–PC line; (2) strip the skull by using iBEAT; (3) identify the remaining non-brain tissues by the boundaries of brain gray matter and manually remove these tissues. The manual correction was performed by two pediatric radiologists with 5 years of experience and differences in identifying the remaining non-brain tissues were resolved by consensus.

#### Calculations of brain region volume

To calculate the volume of each brain region, the preprocessed data were firstly registered to a standard 3D-T1WI-based template (Johns Hopkins University) by linear (rigid transformation) and nonlinear (affine transformation) registrations. And then the volume of each brain region can be estimated by Eq. (1):

1 $$V_{i} = \frac{{\sum\nolimits_{{V_{t} }} {J_{F} } }}{{\left| {{\text{A}}_{3 \times 3}^{\text{rigid}} } \right|\left| {{\text{A}}_{3 \times 3}^{\text{affine}} } \right|}},$$

where *V*_*i*_ is the brain volume of the *i*th region of individual subject, *V*_*t*_ represents the corresponding area in the template. $\left| {{\text{A}}_{3 \times 3}^{\text{rigid}} } \right|$ is the determinant of 3 × 3 sub-matrix in upper left corner of rigid transformation matrix, and $\left| {{\text{A}}_{3 \times 3}^{\text{affine}} } \right|$ is the determinant of 3 × 3 sub-matrix in upper left corner of affine transformation matrix. *J*_*F*_ is Jacobian, for linear transformation, *J*_*F*_ is a constant which is equal to the inverse of the determinant of the 3 × 3 sub-matrix in the upper left corner of the transformation matrix; for nonlinear transformation, *J*_*F*_ is a three-dimensional function.

#### Construction of brain structural network

By using the graph theoretic approaches, the cortical and subcortical regions were used as nodes to construct the brain networks, with connections between nodes defined as correlations between regional brain volumes. Here, 64 brain regions that mainly involved the gray matters and several important subcortical regions (thalamus, hippocampus and cerebellum) were selected as network nodes (Table 1). GRETNA (http://www.nitrc.org/projects/gretna/) was used to construct the network. Partial correlations between all nodes’ volumes were firstly estimated as edges of the network, and then network was constructed by binary connective matrices. The network properties, such as clustering coefficient (*C*_*p*_), characteristic path length (*L*_*p*_), global (*E*_global_) and local efficiency (*E*_local_) were calculated by the following Eqs. (2–5).

**Table 1:** The list of 64 brain regions

No.	Region	Hemisphere	No.	Region	Hemisphere
1	Thalamus	Left	33	Angular gyrus	Left
2	Thalamus	Right	34	Angular gyrus	Right
3	Putamen	Left	35	Superior temporal gyrus	Left
4	Putamen	Right	36	Superior temporal gyrus	Right
5	Globus pallidus	Left	37	Middle temporal gyrus	Left
6	Globus pallidus	Right	38	Middle temporal gyrus	Right
7	Caudate nucleus	Left	39	Inferior temporal gyrus	Left
8	Caudate nucleus	Right	40	Inferior temporal gyrus	Right
9	Superior frontal gyrus	Left	41	Fusiform gyrus	Left
10	Superior frontal gyrus	Right	42	Fusiform gyrus	Right
11	Middle frontal gyrus	Left	43	Parahippocampal gyrus	Left
12	Middle frontal gyrus	Right	44	Parahippocampal gyrus	Right
13	Inferior frontal gyrus	Left	45	Entorhinal cortex	Left
14	Inferior frontal gyrus	Right	46	Entorhinal cortex	Right
15	Medial fronto-orbital gyrus	Left	47	Superior occipital gyrus	Left
16	Medial fronto-orbital gyrus	Right	48	Superior occipital gyrus	Right
17	Lateral fronto-orbital gyrus	Left	49	Middle occipital gyrus	Left
18	Lateral fronto-orbital gyrus	Right	50	Middle occipital gyrus	Right
19	Gyrus rectus	Left	51	Inferior occipital gyrus	Left
20	Gyrus rectus	Right	52	Inferior occipital gyrus	Right
21	Precentral gyrus	Left	53	Cuneus	Left
22	Precentral gyrus	Right	54	Cuneus	Right
23	Postcentral gyrus	Left	55	Lingual gyrus	Left
24	Postcentral gyrus	Right	56	Lingual gyrus	Right
25	Superior parietal gyrus	Left	57	Amygdala	Left
26	Superior parietal gyrus	Right	58	Amygdala	Right
27	Precuneus	Left	59	Hippocampus	Left
28	Precuneus	Right	60	Hippocampus	Right
29	Cingular gyrus	Left	61	Cerebellar hemisphere	Left
30	Cingular gyrus	Right	62	Cerebellar hemisphere	Right
31	Supramarginal gyrus	Left	63	Insular cortex	Left
32	Supramarginal gyrus	Right	64	Insular cortex	Right

The clustering coefficient of a node *i* (C(*i*)) is defined as the likelihood that the neighborhoods of a given node *i* are connected to each other:

2 $$C(i) = \frac{2}{{k_{i} (k_{i} - 1)}}\sum\nolimits_{j,k} {(\varpi_{ij} \varpi_{jk} \varpi_{ki} )}^{1/3} ,$$

where *k*_*i*_ represents the number of edges connected to the node *i*, and $\varpi_{ij}$ is equal to the weight between node *i* and *j*. The clustering coefficient, *C*_*p*_, of a network is the average of the clustering coefficient over all nodes:

3 $$L_{P} (G) = \frac{1}{N(N - 1)}\sum\nolimits_{i \ne j \in G} {L_{ij} } ,$$

where *N* is the number of nodes in the network, and *L*_*ij*_ is the shortest path length between nodes *i* and *j* in a network G. *L*_*p*_ is the average of the shortest path length between all pairs of nodes in the network:

4 $$E_{glob} (G) = \frac{1}{N(N - 1)}\sum\nolimits_{i \ne j \in G} {\frac{1}{{L_{ij} }}} ,$$

where *L*_*ij*_ is the shortest path length between node *i* and node *j* in G, and N represents all nodes in the network:

5 $$E_{loc} (G) = \frac{1}{N}\sum\nolimits_{i \in G} {E_{glob} (G_{i} )} ,$$

where *E*_glob_ (*G*_*i*_) is the global efficiency of *G*_*i*_, the subgraph of the neighbors of node *i*.

The C_*p*_ of a network is defined by the average of the clustering coefficients across nodes, where the C_*p*_ of a node is the ratio of the number of actual connections nearest neighbors of this node to the maximum number of possible connections [31]. C_*p*_ quantifies the local interconnectivity of a graph. The L_*p*_ of a graph is the average of the shortest path length between all pairs of nodes in the network, and it is an indicator of overall routing efficiency of a graph [32]. The *E*_local_ of a network is the average of the local efficiency over all nodes. It measures the mean local efficiency of the network. The *E*_global_ of a network is defined by the mean shortest path length [33]. It measures the extent of information propagation through the whole network. Typically, a small-world network should fulfill the following conditions: C_*p*_/*C*_rand_ > 1 and *L*_*p*_/*L*_rand_≈ 1.

### Statistical analysis

Regarding the manual corrections for anterior commissure–posterior commissure (AC–PC) alignment and skull stripping, the repeatability and consistency of brain volume calculations between two repeated measurements were evaluated by the Bland–Altman graph and intraclass correlation coefficient (ICC). Analysis of variance (ANOVA) was used to compare the differences in the volumes of 64 brain regions between the three workflows.

All the segmentation, calculation of brain region volume and network parameters, and statistical analysis were performed by using the MATLAB R2016b (Mathworks Inc, Natick, MA, USA).

## Data Availability

The codes and datasets of this study are available from the corresponding authors upon reasonable request.
